# Metal-organic frameworks as photocatalysts in energetic and environmental applications

**DOI:** 10.55730/1300-0527.3592

**Published:** 2023-10-11

**Authors:** Elif ÖZCAN, Zeliha MERMER, Yunus ZORLU

**Affiliations:** Gebze Technical University, Department of Chemistry, Kocaeli, Turkiye

**Keywords:** Metal-organic frameworks, photocatalysis, solar energy, pollutant degradation, solar fuel production

## Abstract

Metal-organic frameworks (MOFs) are an exciting new class of porous materials with great potential for photocatalytic applications in the environmental and energy sectors. MOFs provide significant advantages over more traditional materials when used as photocatalysts due to their high surface area, adaptable topologies, and functional ability. In this article, we summarize current developments in the use of MOFs as photocatalysts for a variety of applications, such as CO_2_ reduction, water splitting, pollutant degradation, and hydrogen production. We discuss the fundamental properties of MOFs that make them ideal for photocatalytic applications, as well as strategies for improving their performance. The opportunities and challenges presented by this rapidly expanding field are also highlighted.

## 1. Introduction

Environmental degradation and the energy crisis are two major problems that threaten modern society’s capacity to survive. Due to the fact that solar is a type of sustainable renewable energy source and is also the planet’s most plentiful and readily available clean energy source, solar light-driven advanced technologies are renewable and green environmental restoration methods that have gained popularity for two decades [1]. Photocatalysis is a green and environmentally friendly process in which solar light energy is used to start a chemical reaction [2]. The catalytic reaction occurring in photocatalysis is an advanced oxidation process in which light energy is converted into chemical energy and free radicals as hydroxyl radicals are produced. Of much interest is the potential of degrading pollutants into nontoxic by-products by harnessing solar energy. Due to its benefits, photocatalysis has generated a lot of interest. Here are some benefits of photocatalysis: (i) Environmentally friendly: People are drawn to photocatalytic processes since they do not produce any toxic byproducts or pollutants. This enables the process to be environmentally friendly while having few negative effects. (ii) Energy-efficient: Photocatalysts use light energy as the catalyst for photooxidation reactions. It is well recognized that light energy is a sustainable and clean kind of energy. (iii) Selectivity: With the help of particular photocatalytic reactions, products with high yields and purity can be created. (iv) Photocatalysis can be carried out under mild reaction conditions, such as ambient temperature and pressure. (v) Scability: Industrial applications on a large scale can be simply modified to use the photocatalytic process. (vi) Versatility: Applications for photocatalysis in a variety of fields, such as water treatment, energy conversion, organic synthesis, air purification, and others, have attracted a lot of attention [3,4]. To sum up, photocatalysis is a promising technology that offers a number of benefits over conventional methods. MOF is a crystalline porous material formed by connecting inorganic metal centers and bridging organic ligands through self-assembly. In recent years, an increasing number of researchers have devoted their efforts to the photocatalytic degradation of organic pollutants in water, CO_2_ reduction, H_2_ production, and dye degradation from wastewater by MOFs. Zhang et al. [5] reviewed the latest progress in the photocatalytic degradation of organic pollutants in air and water using functionalized MOFs. Xiang et al. reviewed MOF-based photocatalysts for CO_2_ reduction [6]. Jiang et al. reviewed the progress of MOF-based photocatalysis for solar fuel production [7]. However, these articles have mainly summarized only one or two applications of photocatalyst MOF in the energy and environmental areas. This review systematically introduces the preparation and mechanism of photocatalyst MOFs and focuses on their potential applications. Consequently, we specifically discuss the application of MOFs as a photocatalyst, CO_2_ reduction, water purification, solar fuel production H_2_ production and so on. We hope this review can provide useful guidance for the further development of photocatalysts as a MOFs.

Several recent reviews have delved into the advancements and breakthroughs of MOFs in energy-related applications and catalytic processes [8–10]. These reviews have aimed to provide comprehensive insights into the latest developments, challenges, and opportunities in utilizing MOFs in addressing global energy and environmental concerns [11–15]. Researchers have extensively explored the use of MOFs in energy storage technologies like batteries, supercapacitors, and hydrogen storage, as well as in energy conversion applications such as photocatalysis and solar cells [11–19]. Moreover, MOFs’ catalytic potential in various chemical reactions, including hydrogenation, oxidation, and carbon dioxide conversion, has been the subject of intense investigation [8–15].

Amidst the growing number of reviews on MOFs in energy and catalytical applications, the present review seeks to offer novel perspectives and contributions to the field. Firstly, it provides an updated and critical analysis of the most recent research findings, covering the literature. This ensures that the review incorporates the latest developments in the field, enabling readers to gain a comprehensive understanding of the current state of MOFs.

Secondly, the review uniquely highlights the challenges and limitations faced in the practical application of MOFs in energy and catalysis. While MOFs exhibit remarkable potential in laboratory settings, the transition to real-world applications necessitates addressing issues such as stability, scalability, and cost-effectiveness. By emphasizing these challenges, the review sheds light on the areas where further research and development are required to make MOFs viable for large-scale implementation.

Additionally, the review assesses the environmental impact and sustainability aspects associated with MOFs’ synthesis and usage. As sustainability becomes an increasingly critical aspect of modern scientific research, this novel perspective provides researchers and policymakers with crucial insights into the overall ecological footprint of MOF-based technologies.

## 2. Photocatalysis

Photocatalysis is a photoinduced oxidation process that semiconductor allows the formation of strong oxidants (h^+^) in the valence band (VB) and reductants (e^−^) in the conduction band (CB) by light absorption. Metal oxides, nanomaterials [20], metal (oxy) nitrides, metal phosphides, metal (oxy) sulfides, and carbon-based composites are semiconductors utilized as heterogeneous photocatalysts for directly solar energy-driven photocatalysis. Some characteristics of these heterogeneous photocatalysts include surface modification, recycling, and maximizing photocatalytic activity [1,2,4].

Recently, photocatalytic applications have been facing problems such as photocatalysts that are less stable, low production yields, quantum efficiencies and particularly, slow photocatalytic activity. The wide bandgap, quick recombination of photogenerated electron-hole pairs (EHPs), low solar light energy consumption efficiency, photocorrosive, and poor recyclability are some of the drawbacks of semiconductors, though.

It is vital to develop novel and highly effective photocatalysts that can be controlled to perform various activities activities in order to overcome these challenges. Superior functional materials that exhibit exceptional overall photocatalytic activity appear to be widely desired [4,8].

MOFs, which are also called micro- and meso-hybrid porous crystalline materials, are good candidates for making novel photocatalysts [9]. Inorganic metal clusters and multitopic organic linkers generate coordination bonds to form MOFs, a class of functional inorganic-organic hybrid materials that can be used to create optical, electrical, and catalytic systems [9–11]. MOFs are also known as porous coordination polymers. High surface area, structural designability, tunable pore channels, high surface-to-volume ratios, flexibility to be functionalized with different ligands and metal centers which can be extended up to 90% of the crystal volume, and rich compositions are just a few of the extraordinary features of MOFs structures [8–11]. These distinctive characteristics depict MOFs as porous network architectures, which have drawn significant research interest in a number of potential application areas, including gas storage [12], separation [13], drug delivery [14], sensing [15], nonlinear optics [21], luminosity [22], heterogeneous catalysis [23], photocatalysis [24], and carbon capture and transformation [25,26].

Thanks to the three main active sites in functionalized MOFs, which are active metal sites, organic linkers containing reactive functional groups and external functional groups, the potential of MOFs in heterogeneous photocatalysis occur under UV-Vis irradiation [9,10].

MOFs exhibit semiconductor-like behavior upon light irradiation, that the metal centers can act as single-site catalysts for photocatalysis, and organic linkers can act as antennas to harvest energy and subsequently transfer it to active metal sites. This phenomenon is known as the linker to metal charge transition (LMCT) [8–11].

Many studies have reported the use of different organic ligands and different functional groups on the ligands to adjust the band gap structure of MOFs in order to enhance their visible light absorption and photocatalytic properties. Upon appropriate substitution of electron-donating functional linkers or the incorporation of elongated π-conjugated compounds, it is possible to redshift the visible absorption of MOFs for the efficient utilization of solar energy (i.e. narrowing the band gap of MOFs) [18,27,28].

Defects in MOFs can provide a tunable coordination microenvironment for efficient photocatalysis. Defect engineering presents multiple advantages in manipulating the defect shape, pore size and electronic structure, including the d-band center, charge distribution, and spin moment in MOFs. This is a superior strategy to tune the electronic structure in MOFs further to promote photocatalytic performance [29].

## 3. Metal-organic frameworks (MOFs)

MOFs with a well-defined crystalline structure are a class of highly porous materials that have shown great potential in photocatalytic applications for energetic and environmental purposes [30]. Due to their large surface area and tunable pore sizes, MOFs can be used for various applications [12–15,21–26,31,32]. As photocatalysts, one of the most promising applications of MOFs is in the fields of energy conversion [33]. The hydrogen produced with photocatalysis can be used as a clean and renewable energy source [34]. Also, one of the much more studied field MOFs as photocatalysts is water splitting [35,36]. Of much interest is the potential of water splitting by photocatalysts, which can be used to split water molecules into hydrogen and oxygen [35].

One of the interesting application fields of MOFs is environmental remediation [37]. By exploiting the light source to break down pollutants into non-toxic chemicals, MOFs have the potential to be employed to reduce organic pollutants by photocatalytic degradation [38]. MOFs have been investigated for their potential in the photocatalytic degradation of pollutants such as organic dyes and organic compounds via UV irradiation in the application field of the environment [38–41]. With this clean energy application, it may be possible to remediate dirty water and air in a way that is both affordable and environmentally benign [1,3,42]. CO_2_ capture and utilization is also an application of MOFs as photocatalysts. CO_2_ is captured from industrial processes and converted it into useful products as fuels or chemicals via photocatalytic CO_2_ reduction [27,43]. As a whole, MOFs have great potential as photocatalysts in various energy and environmental applications, and research in this area is expected to lead to forward advancements in the field to optimize their performance and develop practical applications.

**Figure 1:**
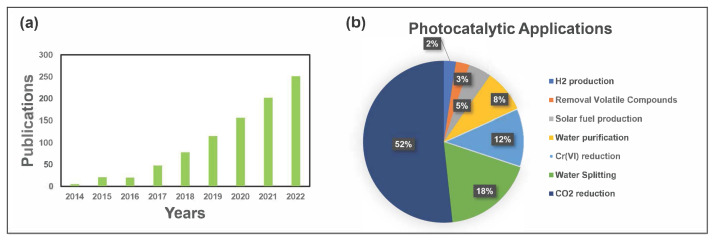
(a). The number of photocatalytic MOF publications per year (2014–2022). (b) Publications percent of MOFs for photocatalytic applications areas. Data obtained from the Web of Science as of April 2023.

The rising number of publications per year in the domain of MOFs’ use in energy and environmental applications is evidence of the growing interest in the subject (Figures 1(a) and 1(b)). By the end of 2023, it is projected that there will have been about 300 papers on photocatalytic MOF. Additionally, MOF-based materials have been used for the removal of heavy metals [44,45], the recovery of precious metals [46,47], the capture of CO_2_ and other toxic gases [48,49], water splitting [35,36], production of solar fuel [50–52], and photocatalytic degradation of various organic and inorganic pollutants [38–42] (Figure 1b).

MOFs can be engineered to display semiconducting properties by adjusting their structure and composition. Particularly, it has been discovered that some MOFs exhibit visible light band gaps, making them potential candidates for use in photocatalysis, solar cells, and other optoelectronic devices [1–4, 8]. The existence of inorganic clusters or metal centers inside the framework, which can function as charge carriers and take part in electron transfer activities, is frequently cited as the cause of MOFs’ semiconducting characteristic.

**Figure 2:**
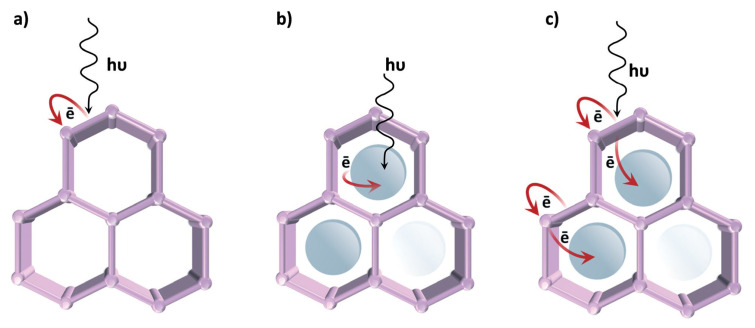
Approaches for photocatalytic activity in MOFs: a) the organic linker harvests the light and LMCT is promoted; b) the MOF is used as a container of a light absorbing catalyst; c) charge transfer occurs between the MOF and the encapsulated catalyst [51].

By selecting the proper metal ions or ligands and managing the synthesis conditions, these clusters or metal centers can be selectively inserted into the MOF structure [1–4, 8]. Additionally, the organic ligands can be functionalized with electron-donating or electron-withdrawing groups to alter the electronic characteristics of MOFs, or the MOFs can be doped with guest molecules. This can change the band gaps and band locations of MOFs, making them better suited for particular applications [9–12]. MOFs offer a number of methods for producing photocatalytic activity, as opposed to conventional semiconductor materials, which frequently rely on surface modification by noble metal nanoparticles or transition metal complexes to produce tunability (Figure 2) [51].

One approach is to employ LMCT, using the organic linker as an antenna for light sensitization and charge transfer to the inorganic cluster. This produces effective charge separation, which can be further improved by adjusting the organic linker by adding more substituents, using mixed linkers, or capping more metal ions. While the optical response can be changed by modifying the cluster-forming metal or employing mixed metal clusters, the reductive power can also be changed by choosing metal ions with the appropriate orbitals. Overall, MOFs are attractive materials for a variety of optoelectronic applications due to their unique advantages as semiconductors, such as their enormous surface area, porosity, and tunable electronic characteristics [1–4,8–12,17,51].

MOFs were generally used as efficient photocatalysts due to their features such as functional organic linkers, the large surface area and permanent porosity, the richness of metal-containing secondary building units (SBUs) as catalytically active sites, and a tuneable band gap [9–12,51].

MOFs have garnered significant attention as potential photocatalysts in energy and environmental applications. Below are some advantages and disadvantages of using MOFs in these areas, focusing on their role as photocatalysts:

**Advantages of MOFs in energy and photocatalytic applications:**

High surface area: MOFs typically possess an extensive surface area and tunable pore sizes, allowing for enhanced adsorption of reactants and efficient utilization of active sites during photocatalysis. This feature enhances their photocatalytic efficiency.

Tailorable properties: The structure and properties of MOFs can be customized through the selection of metal nodes and organic linkers, enabling the design of photocatalysts with specific properties to target different energy and environmental applications.

Versatility: MOFs can be utilized for a wide range of photocatalytic reactions due to their diverse metal nodes and organic linkers. This versatility enables their use in applications such as water splitting for hydrogen production, CO_2_ reduction, and organic pollutant degradation.

Stability: Some MOFs exhibit excellent chemical and thermal stability, which is essential for long-term photocatalytic performance, especially under harsh reaction conditions.

Light harvesting: MOFs can be engineered to absorb a broad range of light wavelengths, including visible light, which makes them efficient photocatalysts for solar-driven energy and environmental processes.

**Disadvantages of MOFs in energy and photocatalytic applications:**

Limited practical applications: Despite their potential, the practical implementation of MOFs as photocatalysts in large-scale energy and environmental processes is still limited. Challenges such as scalability, cost-effectiveness, and stability under prolonged operational conditions need to be addressed.

Mass transfer limitations: Some MOFs may have restricted diffusion properties, which can lead to limitations in the mass transfer of reactants and products, hindering overall photocatalytic efficiency.

Photocorrosion: In certain cases, the presence of certain metal nodes in MOFs may lead to photocorrosion under prolonged light exposure, compromising the stability and effectiveness of the photocatalyst.

Synthesis complexity: The synthesis of MOFs can be complex and time-consuming, and the precise control of their structure and properties can be challenging. This complexity may hinder their widespread adoption in practical applications.

Recyclability: The ease of catalyst recovery and recyclability is a crucial factor for practical photocatalytic applications. Some MOFs may face challenges in regeneration and reusability, impacting their overall sustainability.

In conclusion, MOFs show great promise as photocatalysts in energy and environmental applications due to their unique properties and tunability. However, overcoming challenges related to scalability, stability, and cost-effectiveness will be essential for their successful integration into practical and large-scale applications.

### 3.1. Synthesis of MOFs

**Figure 3:**
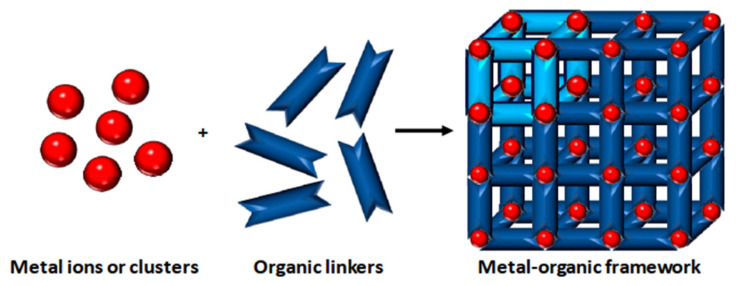
Scheme for the preparation of a MOF [53].

MOFs are synthesized using structurally rigid organic linkers to connect metal building blocks through coordination bonds, resulting in a high-crystalline structure with a symmetrical shape and confined geometrical arrangement (Figure 3) [53]. The strength of these bonds is crucial in establishing the physicochemical properties of the structure. The porous structure of MOFs can be adjusted by geometrical fine-tuning between metal ions and organic linkers, and functional groups can be added during the activation process to alter their functional behavior [54].

MOF synthesis involve using various techniques as solvotermal synthesis, hydrotermal synthesis, and microwave-assisted synthesis. The choice of synthesis technique depends on the desired properties of MOFs and the scale of production. MOF synthesis involves the combination of metal ions with organic ligands to form a highly porous material with a crystalline structure. The process typically involves the following steps: (i) Selection of metal ions or metal clusters: depends on the desired properties of MOF. Common divalent metal ions used in MOF synthesis are copper, zinc, and nickel. (ii) Selection of organic ligands: Organic ligands coordinate with metal ions and form stable bonds. Common ligands used in MOF synthesis are structurally rigid molecular building blocks derived from carboxylate, phosphonate, pyridine and imidazole. (iii) Mixing of metal ions and organic ligands: The components are mixed in a suitable medium, such as water or an organic solvent, under controlled conditions of temperature and pH. (iv) Crystallization: The mixture is allowed to crystallize under conditions of temperature and pressure to form a MOF structure. (v) Postsynthesis treatment: It is needed to improve the properties of MOFs, as thermal stability and porosity [16–19,28–29,31–32,53–56].

**Figure 4:**
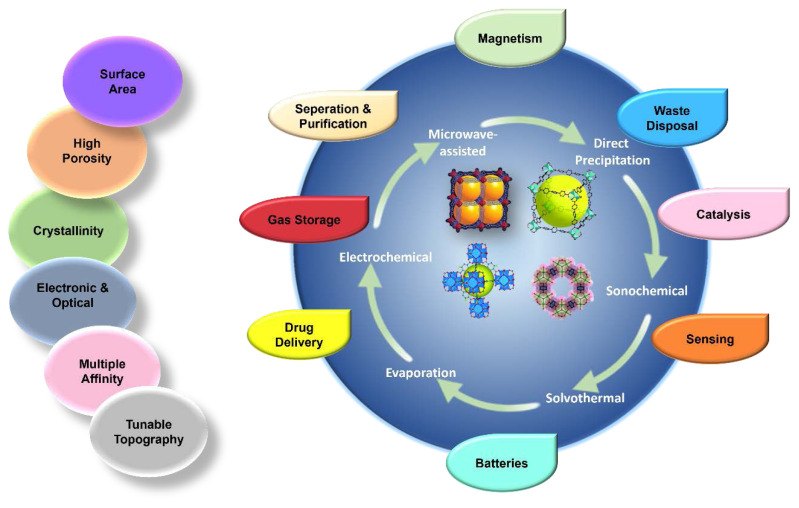
A schematic view of MOF synthesis, properties, and applications.

MOFs are a group of organic-inorganic hybrid units that self-assemble to form a porous, periodic structure with distinctive structural topologies, large surface areas, and exceptionally high porosity. They are used in a wide variety of processes, including catalysis, proton conduction, gas storage, medication delivery, sensing, and separation [12–15, 21–26]. Researchers have improved MOFs’ characteristics by adding metal clusters, organic ligands, and visitors with functional sites using a variety of functionalization techniques, such as postsynthetic modification [30,53–56]. MOFs are the ideal platform for designing and producing functionally manufactured materials due to their multi-functional sites. Figure 4 depicts the various synthesis methods, noteworthy MOF properties, and uses of MOFs.

### 3.2. Characterization of MOFs

MOFs have received a great deal of attention due to their potential applications in a variety of fields, such as gas storage, separation, and catalysis [12,13,24]. Understanding MOF characteristics and customizing their structures for particular applications are crucial [9]. Thermogravimetric analysis (TGA), fourier-transform infrared spectroscopy (FTIR), scanning electron microscopy (SEM), transmission electron microscopy (TEM), N_2_ adsorption-desorption isotherms, and X-ray diffraction techniques (single crystal/powder diffraction) are common methods for characterizing MOFs [5].

The crystal structure and crystallinity of MOFs can be determined by XRD, while SEM and TEM can be used to visualize their morphology and particle size. The functional groups that are present in MOFs can be identified using FTIR, and their thermal stability can be determined using TGA. Surface, pore size, and pore volume of MOFs can be calculated using N_2_ adsorption-desorption isotherms. Overall, these methods of characterizing materials can aid in the logical design of MOFs with tailored properties for particular uses. MOFs are characterized using UV-Vis. Diffuse Reflectance Spectroscopy (UV-Vis. DRS). This method evaluates a solid sample’s absorption spectra as a function of wavelength over the UV-Vis. region of the electromagnetic spectrum (200–800 nm). In UV-Vis. DRS, light is directed at the sample surface, and the amount of light reflected from the surface is measured. The intensity of the reflected light gives information about the electronic transitions that are taking place within the MOF. Specifically, the UV-Vis. DRS spectrum provides information on the band gap energy of the MOF, which is related to the electronic structure and properties of the material. The technique is useful for investigating the optical and electronic properties of MOFs, which are important for their potential applications in catalysis, sensing, and other areas [9,57]. Since many MOF applications, such as water purification, catalysis, and drug delivery, call for their use in aquatic environments, aqueous stability testing is crucial for MOFs. MOFs are porous materials that can adsorb water; however, water exposure can also cause the framework structure to disintegrate, resulting in the loss of specific MOF capabilities. To guarantee their effectiveness and lifespan in various applications, it is crucial to ascertain the stability of MOFs in aqueous settings. In order to maximize the performance of MOFs in certain applications, aqueous stability testing can also offer information on the variables that affect MOF stability, including pH, temperature, and ionic strength [57].

## 4. Water purificiation by photocatalysis

A significant issue on Earth is the lack of access to safe, clean, and quality water, which has an impact on billions of people, living things, and the environment. The key elements that have a direct impact on water quality are pollution, climate change, excessive groundwater exploitation, and inadequate infrastructure. Agricultural, industrial, and urban services all contribute significantly to poor water quality. Water supplies can become contaminated with diseases, toxic chemicals, and heavy metals, rendering them unsuitable for human consumption. Pesticides and agricultural fertilizers can potentially contaminate the water. The second issue is climate change, which exacerbates issues with water quality. Sustainable water management strategies must be used as water purification techniques to address these issues. All people can have access to safe and clean water by improving infrastructure for wastewater treatment and purification technology [57–59].

The removal of organic and inorganic contaminants from water using MOF as a photocatalyst is a promising method. An example of a porous substance is a MOF, which is made up of organic molecules connecting metal ions or clusters to form a crystalline structure with a large surface area [1–3,42]. By adding semiconductor nanoparticles or light-absorbing molecules, MOFs can improve their photocatalytic activity. These substances form electron-hole pairs when exposed to light, which can then interact with water molecules to create reactive oxygen species (ROS) such as hydroxyl radicals (•OH), which can oxidize and destroy contaminants. Titanium dioxide (TiO_2_), which has a high photocatalytic activity and is quite stable under photocatalytic conditions, is one popular photocatalyst used in water purification. TiO_2_ can only be activated by UV light and has a wide bandgap (3.2 eV), which restricts its usefulness. Researchers have created MOFs that contain additional semiconductor components that can be triggered by visible light, such as copper(I) oxide (Cu_2_O), cadmium sulfide (CdS), and zinc oxide (ZnO), in order to get around this restriction. These substances have been demonstrated to efficiently remove organic contaminants from water, including colors, phenols, and pesticides. The high surface area, variable pore size, and chemical stability of MOFs are further benefits for water filtration in addition to their strong photocatalytic activity [23–26,30]. MOFs are relatively simple to synthesize and can be altered to improve their photocatalytic capabilities for certain uses. Overall, the purification of water using a photocatalyst in a MOF is a promising method that may be able to meet the increasing global demand for clean water. To maximize the photocatalytic activity and stability of MOFs, however, as well as scale up the technology for real-world uses, more study is required [23–26,30,38–42,57].

**Figure 5:**
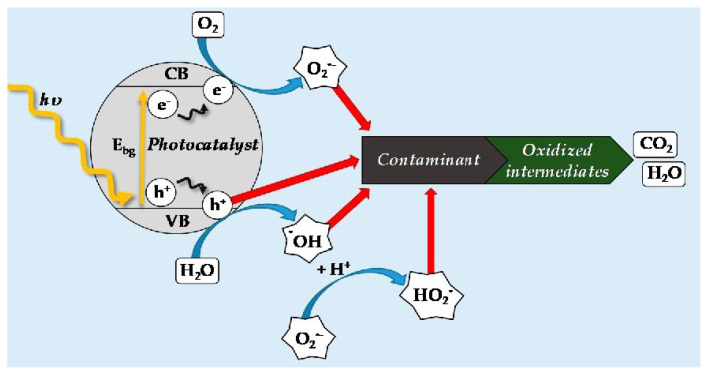
The schematic representation of MOF acting as a photocatalyst in an aqueous solution [57].

A photocatalyst is subjected to light (usually UV or visible light), for example, MOF photocatalyst. Light energy is absorbed by photocatalytic species (such as semiconductors), which produce electron-hole pairs. Separated electrons (e^−^) and holes (h^+^) move to the MOF’s surface. At the MOF’s surface, electrons and holes can take part in redox reactions with water molecules or contaminants that have been adsorbed [1,3, 23–26,30,57]. In the presence of water (H_2_O), electrons (e) can reduce oxygen molecules (O_2_) to produce superoxide radicals. Water molecules can be oxidized by holes (h^+^) to produce hydroxyl radicals (•OH). Highly reactive oxidant species such as superoxide radicals and hydroxyl radicals (•OH) can oxidize or destroy contaminants by a variety of processes, including electron transfer, radical reactions, and hydroxyl radical attack. Pollutants that come into contact with the reactive oxidant species produced by the photocatalyst are oxidized or degraded into innocuous products (e.g., organic dyes, medicines, pesticides, heavy metals, and microorganisms) (Figure 5) [35,38–42,44–47,57].

Pollutants in the aqueous solution can continue to be broken down as long as the MOF photocatalyst is exposed to light since it can keep producing reactive oxidant species. MIL-101(Cr), constructed from chromium ions and a terephthalic acid ligand, is an example of a stable MOF used for water purification. Heavy metals, organic dyes, and medicines can all be removed from water using MIL-101(Cr) because of its high surface area and variable pore size [57,60,61]. A few of the most extensively investigated MOFs include ZIF-8, UiO-66(Zr), MIL-125(Ti), MIL-101, MIL-53, and MIL-88B [62], which employ light to transport photogenerated electrons for the treatment of water through photocatalysis [63,64]. In order to degrade Rhodamine B (RhB) under visible light (550 nm), Laurier et al. described the photocatalytic activity of different Fe-MOFs (MIL-88B, MIL-100, and MIL-101) [65]. In order to find more stable and active photocatalysts, MOF synthesis and modification have significantly advanced. Numerous MOFs have been identified in the literature for photocatalytic water treatment, and subsections have been developed to investigate their efficacy on various pollutants, including dyes, heavy metals, priority, and emergent contaminants [57,60,61,63–65].

### 4.1. Photodegredation of dyes

Due to their mutagenic and carcinogenic effects, dye effluents from a variety of industries, including textiles, plastics, rubber, paper, cosmetics, leather, and printing, can seriously harm aquatic organisms and people [66,67]. Considering that the majority of dyes are persistent and nonbiodegradable, conventional biological treatments are frequently ineffective at removing colors. Numerous MOFs are being studied as photocatalysts for the breakdown of organic dyes in wastewater, and photocatalysis has emerged as a potential approach for their removal. However, rather than real dyestuff water, much research has only concentrated on the removal of commercial colors in synthetic fluids. Due to their simplicity of analysis, methyl blue (MB), methyl orange (MO), and rhodamine B (RhB) are frequently targeted pollutants [6,57,66,67]. For the removal of these dyes from water, photocatalysis has emerged as a potential method, and MOFs have been demonstrated to be efficient photocatalysts in this application. In order to build and optimize more effective and selective photocatalysts for water treatment applications, it is important to understand the mechanisms and variables influencing the photodegradation of dyes. The fundamental reason why UV light is used more frequently than visible light and sunlight is because pure MOFs show relatively high band gap values [6,57,66,67]. To speed up the degradation rate, additional oxidants such as H_2_O_2_ or persulfate may occasionally be required. Table 1 presents pertinent illustrations of some MOFs for dye degradation, organized by the type of radiation used.

**Figure 6:**
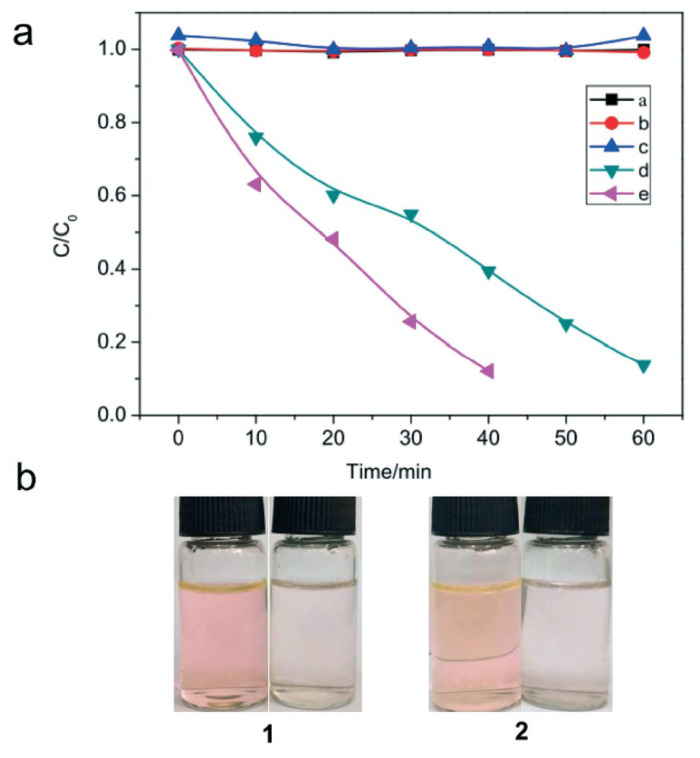
The experiment studied the degradation of R6G (a dye) in a solution under various conditions, comparing the results with and without light irradiation. The different conditions tested were as follows: (a) R6G/UV light without a catalyst (denoted as —■—a). (b) R6G in the dark with compound 1. (c) R6G in the dark with compound 2. (d) R6G with compound 1 under UV light. (e) R6G with compound 2 under UV light [81].

**Figure 7:**
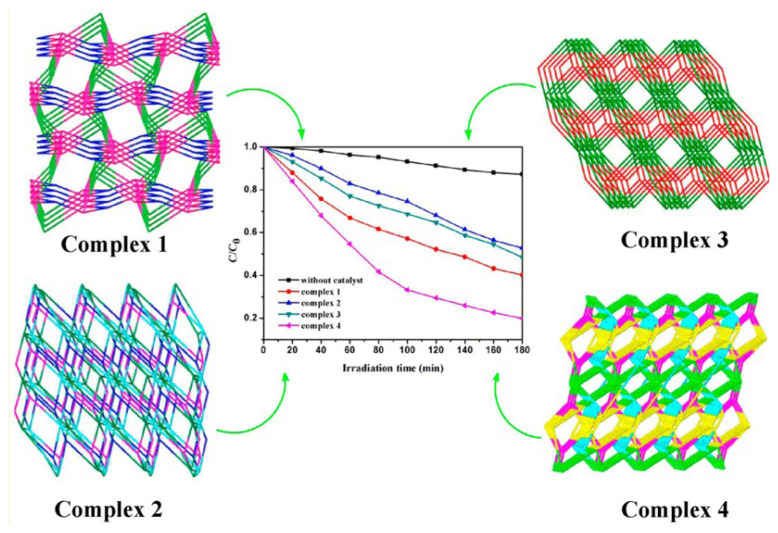
Crystal structures of Cd (II) complexes (1–4) and the photocatalytic degradation of MB and MO solution under UV-light irradiation 1–4 [82].

Zhang et al. tested Cd(II)-imidazole MOFs for MO degradation and showed that activity was not only controlled by the adequate band gap, but other factors such as efficiency in charge transfer and separation should be taken into account [79]. Due to the excitation of the Zn-complexes, ZIF-8 also displayed strong activity for MB breakdown under UV light [80]. According to a publication in [81], Ag(I)-tetrazole MOFs have an appropriate ligand for the creation of active photocatalysts for the degradation of Rhodamine R6G (Figure 6). Higher degradation rates were achieved by favoring the linker-metal cluster charge-transfer process by using ligands with more delocalized electrons, such as ethylenediamine and 5,5′-tetrazolate [57]. To test the impact of polynuclear complexes on photocatalytic dye degradation, Liu and colleagues recently produced coordination complexes made of Cd(II) clusters (Figure 7) [82]. The complexes were created by heating a mixture of Cd(NO_3_)_2_.4H_2_O, ligands, ethanol, and water in a stainless steel reactor while using auxiliary ligands of hbmb=1,1′-(1,6-hexane)bis(2-methylbenzimidazole), btbb=1,4-bis(2-(4thiazolyl)benzimidazole-1-ylmethyl)benzene, 4,4′-bipy = 4,4′-bipyridine).

Under UV light irradiation, the four resultant polynuclear Cd(II) complexes were employed to break down MB and MO, and the rates of degradation were assessed. The research discovered that the MO degradation process benefited from the cooperation of dinuclear and tetranuclear Cd (II) clusters in one of the complexes [82]. The generation of hydroxyl radicals was impacted by the MB and MO dyes’ various structural differences, which led to varying rates of degradation. According to the study, cluster complexes with various nuclei may have various effects on how organic dyes degrade [67,82]. Designing active MOFs for photocatalysis depends critically on selecting the right metal clusters. The ligands are also essential to the photocatalytic activity for dye degradation, even though some metals’ d-d spin can cause MOF absorption to shift to the visible spectrum. The synthesis of stable and useful MOFs typically involves the use of N,O-donor and N-containing ligands. Using innovative Cu-MOFs with a combination of N,O-donor ligands (pyridine- and imidazole-based ligands), Qiao et al. reported the degradation of multiple dyes (MO, MB, and RhB) under visible light, reaching roughly 70%–80% degradation in less than 4 h [83]. Using a Co-MOF built up by a triazaindolizine-based linker, the entire MB conversion was completed in 2.5 h. Although lone electron pairs in the structure of N-donor ligands were also chosen because they can be swiftly transported to the metal clusters, their photocatalytic performances fell short of expectations [83]. Surprisingly, this kind of catalyst displayed greater activity under UV light, demonstrating that other factors should also be taken into account when designing visibly active photocatalysts [57,83]. Based on these N-donor ligands, it has been investigated how known MOFs can be altered by including -NH_2_ in the ligand [57,83]. Its inclusion typically causes the band gap to close, expanding the absorption of light into the visible spectrum. Despite having tiny band gaps, the photocatalytic activity of NH_2_-MOFs for dye removal did not stand out. Despite having a smaller band gap than UiO-66, Mu et al. showed that UiO-66-NH_2_ exhibited inferior photocatalytic performance for RhB degradation than UiO-66 [57,84].

### 4.2. Photocatalytic heavy metal removal and Cr(VI) reduction

**Figure 8:**
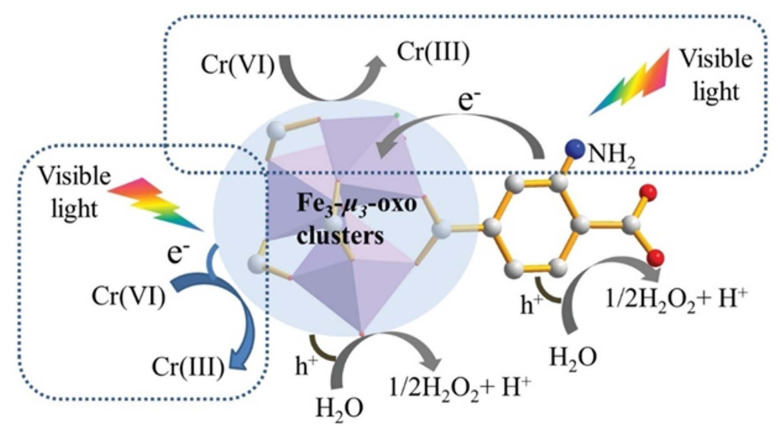
Mechanism of excitation pathways for photocatalytic reduction of Cr(VI) over NH_2_–MIL-88B (Fe) [94].

Heavy metals play a key role in the toxicity of pollutants that need to be eliminated, making water contamination a big concern. Surface water and industrial wastewater have been discovered to contain a number of heavy metals, including lead, mercury, cadmium, arsenic, and chromium, which are acutely harmful to both people and aquatic life [7,20,54,57,85–88]. Heavy metals are metallic elements that have historically been used by people and have the potential to negatively impact both the environment and human health [7,20,54,85–88]. The metallic element chromium can be found in hexavalent form, commonly known as Cr(VI), where the chromium atom is in the +6 oxidation state. It is a widely used chemical that is extremely hazardous and carcinogenic, and it is utilized in many industrial operations such as welding, electroplating, and chromate painting [20,54, 57,87–89]. Governments and international organizations regulate Cr(VI) due to its hazardous and carcinogenic qualities. In order to make chromium less poisonous and detrimental to the environment, chromium is reduced from its Cr(VI) oxidation state to Cr(III) oxidation state. Cr(VI) is a highly poisonous substance that, when consumed or inhaled, can result in serious health issues. It is also a significant environmental pollutant [7,20,54,86,87]. Because Cr(VI) is highly hazardous and carcinogenic, this method is frequently employed in industrial and environmental applications to remove Cr(VI) ions from wastewater. A range of techniques, including chemical, electrochemical, and biological methodologies, are utilized for the purpose of reducing Cr(VI) in aqueous solutions. It can be employed in a variety of industrial applications and safely disposed of after being reduced from Cr(VI) to Cr(III). To prevent further environmental contamination or harm to human health, it is crucial to make sure that the reduction process is carried out safely and that the resulting trivalent chromium is handled appropriately [10,57,87,88]. When Cr(VI) is transformed into Cr(III), it loses some of its solubility and toxicity, which makes it simpler to remove from the environment. Because of its expanding use across a number of industries, including tanneries and metal plating, the remediation of Cr(VI) is essential. MOFs have recently been investigated for their potential to convert Cr(VI) to Cr(III). MOFs offer a lot of surface area for adsorbing Cr(VI) from solutions owing to its porous nature, and the metal ions they contain can function as reducing agents to turn Cr(VI) into Cr(III). Although the exact process of Cr(VI) reduction in MOFs is still not known, it is thought to include the transfer of electrons from the MOF to the Cr(VI) species [7,20,54,87,88]. Functional groups on the MOF surface may play a role in electron transfer [57, 7,20,54,87,88], and the MOF’s porous structure provides a large surface area for interactions with Cr(VI) species. For the reduction of Cr(VI), a variety of MOFs have been investigated, including MIL-101, UiO-66, and ZIF-8 [89–92]. Under diverse circumstances, these MOFs have demonstrated remarkable efficacy in converting Cr(VI) to Cr(III) [57,89–92]. According to one study, MIL-101 and UiO-66 both demonstrated significant Cr(VI) removal efficiencies of up to 99.9% and 97%, respectively [89,91]. According to another investigation, ZIF-8 may efficiently reduce Cr(VI) to Cr(III) with up to 99.9% efficiency [92]. One of the composite MIL-100(Fe) performances, HPMo@MIL-100(Fe), just took eight min to reduce Cr(VI) efficiently [93]. The performance of a few MOFs and their composites as photocatalysts for the reduction of Cr(VI) when exposed to visible light is listed in Table 2. The catalytic activity is also impacted by MOF functionalization [57]. The photocatalytic performance of MOFs can be enhanced by adding an amine (NH_2_) group [94]. Shi and colleagues created the (NH_2_)-functionalized MOF NH_2_-MIL-88B (Fe), which served as a nice illustration of how adding an amine group to the MIL-88B (Fe) structure might encourage the transfer of electrons [94]. Because the amine group can promote both the electron transfer from the excited amine functionalized organic linker to oxo-metal clusters and the direct excitation of the oxo-metal cluster, the amine (NH_2_)-functionalized MOF displayed an additional absorption band in the visible range [94]. In conclusion, NH_2_-MIL-88B (Fe) exhibits greater stability and efficiency for the photocatalytic reduction of Cr(VI) under visible light irradiation than NH_2_-MIL-88B, indicating that the electron transfer is crucial for boosting the photocatalytic activity as it can lessen the likelihood of electron-hole pairs recombination (Figure 8) [94].

Overall, the application of MOFs for Cr(VI) reduction shows potential as a tool for environmental remediation in a variety of industrial and environmental settings. However, more research is required to improve the conditions for Cr(VI) reduction and to comprehend the mechanism that drives the reduction process. Additionally, great consideration must be given to MOFs’ potential toxicity to the environment and to human health, as well as their stability in various situations.

### 4.3. Photodegradation of priority pollutants

**Figure 9:**
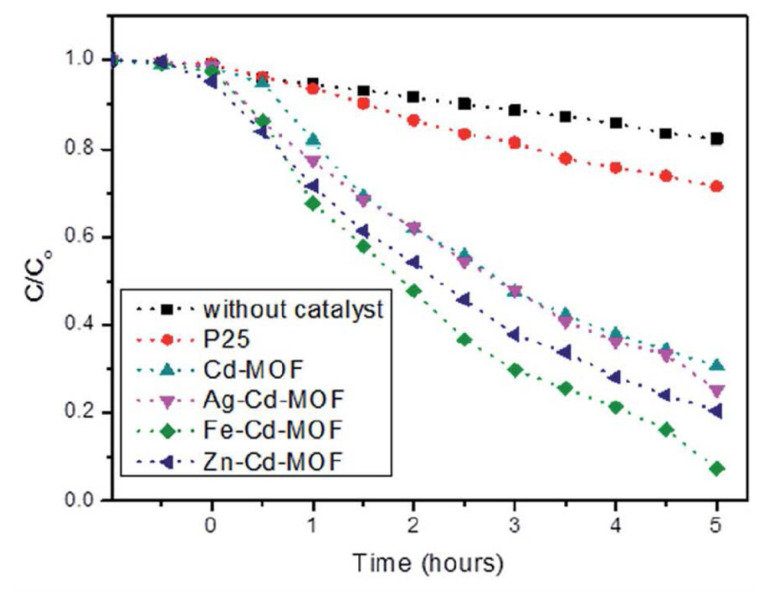
(a) Photocatalytic performance of various MOFs activated under daylight [108].

Toxic chemicals such as pesticides, phthalates, volatile organic compounds, chlorobenzenes, polycyclic aromatic hydrocarbons, and alkylphenols are examples of the priority pollutants found in urban areas and wastewater systems [107–110]. Their toxicity is caused by environmental persistence and carcinogenic and mutagenic effects on people and animals [57]. Concerns about phenol and similar phenolic chemicals have led to a great deal of research in water purification-related technologies. While MOFs have demonstrated promise in the photodegradation of priority pollutants, they remain insufficiently utilized in this field. While phenol and similar compounds have been studied for their ability to degrade using MOFs, their conversion rates have not always been as high as those obtained with other photocatalysts like TiO_2_ and ZnO semiconductors [110]. Recent research, however, suggests that modified MOFs, which include metal ions like Ag^+^, Fe^3+^, and Zn^2+^ intercalated into them, may be more efficient than typical photocatalysts like TiO_2_ [57,108]. In the elimination of 2-chlorophenol under direct sunlight, Surib et al. showed that modified MOFs intercalated with Fe^3+^ achieved a conversion rate of 93% after 5 h [108] (Figure 9). Alvaro et al. also evaluated the activity of MOF-5 for phenol degradation under UV light and attributed this activity to the new energy levels that were induced between the bands of the MOF by the metal ion, lowering the band gap and increasing light absorption. The activity of MOF-5 was higher per metal atom [109]. Even with these encouraging findings, there is still much to learn about the use of MOFs in the degradation of priority contaminants, and additional research is essential to truly understand their potential in this field.

#### 4.3.1. Photocatalytic removal of volatile compounds

The primary causes of air pollution are volatile organic compounds (VOCs), which can disrupt the ozone layer and cause irreparable harm. Chemicals known as VOCs are found in a variety of products, such as paints, cleansers, adhesives, and even some construction materials [111,112]. When people are exposed to them, they can impair their health and contribute to air pollution [111–116]. Some reported VOCs and their related hazards are listed in Table 3 [111]. Adsorption and catalysis are two purification technologies that have been developed recently as a result of strict limits on VOC emissions [111–116]. However, photocatalysis, thermal catalysis, and plasma catalysis have promise; they are not perfect, and adsorption may cause secondary pollution [111,116]. These are mostly studied by photocatalysis and thermocatalysis, with photothermal catalysis being a new area of interest due to their synergistic properties. Catalytic degradation technology requires low-cost, high-performance catalysts and better catalytic reaction process understanding [111–114]. VOCs can be harmful to the environment and people’s health since the atmosphere constantly undergoes photochemical reactions [111]. Therefore, it is essential to do research on the use of VOC photocatalytic degradation for reducing air pollution. Photocatalytic oxidation, a green purifying method, combines semiconductors and light to convert organic molecules into CO_2_ and H_2_O. Due to their capacity for oxidation-reduction under light, MOFs and their derivatives are good pollutant degradation catalysts. A potential field of study for converting solar energy into renewable energy and cleaning up environmental pollution is photocatalysis technology. As catalysts for the degradation of VOCs, MOFs have been the subject of substantial research [111–115]. Table 4 provides an overview of recent research on the effectiveness of photocatalysts made from MOFs and those derived from them.

Toluene is a chemical that is frequently used in studies on gas-phase photocatalytic oxidation, which could produce secondary aerosols in the environment. Photocatalysis is an efficient way to degrade toluene, which is necessary to solve atmospheric problems [111]. Researchers used an in-situ production method to develop an UiO-66 composite material for photocatalytic toluene degradation. The hydrophilic porous reticular UiO-66 encapsulating layer improved the photocatalytic activity, charge transfer, and visible light absorption of UiO-66. In order to increase the photocatalytic activity of composites, the researchers found that a variety of strategies might be applied, such as structural modifications, dopant modifications, and heterojunction structures [123,124].

Other studies using other composites, including TiO_2_ and MIL-101-MOF composites, have shown improved photocatalytic efficacy through better charge transfer and the formation of heterojunctions. The mixture of holes and oxygen-containing free radicals efficiently destroyed toluene with a conversion rate of 91.4% after 480 min of processing [111,119,125]. The development of a highly effective photocatalyst for the selective hydroxylation of benzene to phenol is also crucial. Out of all the MOF-based photocatalysts that Wang et al. have developed, Fe-based MOFs are considered to be the best suitable for this application due to the presence of iron-oxygen clusters and the ease of hydroxyl radical activation. According to research, the iron complexes MIL-100 (Fe) and MIL-68 (Fe) preferentially hydroxylate benzene to produce phenol when exposed to visible light and H_2_O_2_ as an oxidant [126]. This method of benzene hydroxylation is low-cost, long-lasting, and ecologically friendly.

### 4.4. Photodegradation of emerging pollutants

**Figure 10:**
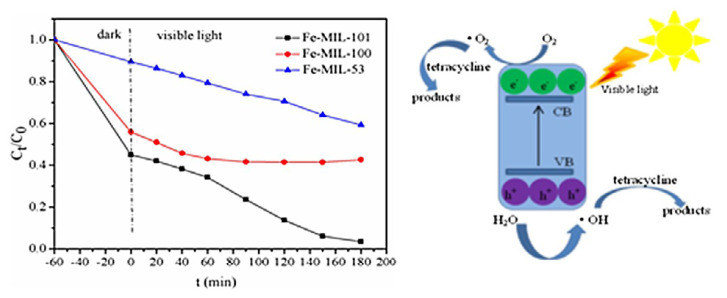
Schematic representation of Fe-based MILs for degradation of tetracycline [129].

Pharmaceuticals, personal care items, and endocrine-disrupting substances are just a few of the chemical substances that have been identified as emerging pollutants in a variety of environmental matrices, including surface water, groundwater, and wastewater [57,127,128]. There is a rising demand for effective and environmentally friendly solutions for the removal of these developing contaminants because of the potential threats they bring to both human health and the environment. Due to the special characteristics of MOFs, including their large surface area, variable pore size, and various metal coordination environments, which enable efficient adsorption and photocatalytic destruction of a wide spectrum of contaminants, photocatalysis utilizing MOFs has emerged as a viable technique. According to recent studies, MOFs can effectively degrade developing contaminants such as antibiotics, dyes, and endocrine-disrupting substances by photocatalytic degradation. For instance, according to certain research, tetracycline is degraded by MOFs like MIL-53, MIL-101, and MIL-100 when exposed to visible light [57,129,130]. As shown in Figure 10, all MOF series are effective for the degradation of tetracycline.

Due to its structure, MIL-101 was determined to be the most effective, yet photocatalysis could only remove 40% of the tetracycline. Modified MOFs, such as metal-ion-doped or functionalized MOFs, have also been investigated in other studies as a means of improving photocatalytic activity. MIL-100 and Pd@MIL-100 have also been tested for the PPCP degradation, however higher conversions than 60% required additional oxidants, including H_2_O_2_ [130]. Ibuprofen degraded effectively under visible light, with a new MOF composite made of Ag/AgCl@MIL-88A(Fe) achieving complete elimination and a 90% drop in TOC after 3 h [131]. Overall, while still in its infancy, the use of MOFs for the photodegradation of emerging contaminants has great promise as a long-term and effective method of removing these dangerous substances from the environment.

## 5. Water splitting by photocatalysis

**Figure 11:**
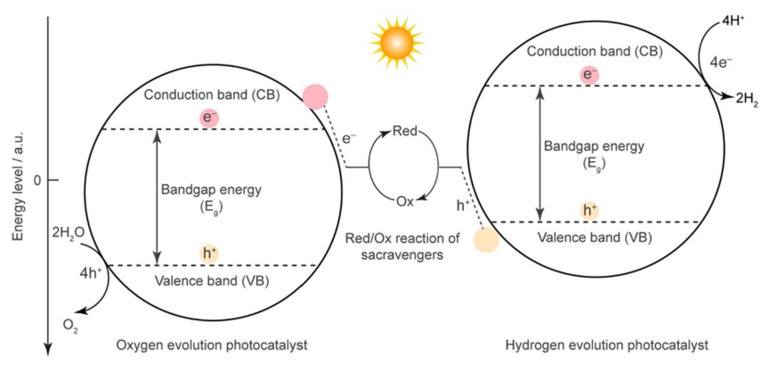
The photocatalytic water-splitting process and its working principle [36].

To solve the world’s energy dilemma, nations all over the world have made switching to renewable energy as a replacement for fossil fuels a top priority. As long as we continue to rely on fossil fuels, it will be impossible to achieve zero or low carbon emissions during this transition [51,57]. Two of the seven forms of renewable energy regarded as the energy of the future are hydrogen and solar [51]. In contrast to hydrogen, which must be manufactured industrially through procedures like the steam reforming of hydrogen carbons (such as methane), which uses a lot of energy and has negative environmental effects, solar energy is abundant and accessible everywhere [51,52,57]. But if water, a plentiful natural resource, can be artificially divided into hydrogen and oxygen via photocatalytic water splitting powered by solar energy, it might lead to an endless supply of hydrogen [36,57]. This procedure would photocatalyze water into dihydrogen and dioxygen using solar energy, making it the ideal use of solar energy. By dividing the water molecules, the process known as “water splitting” (2H_2_O → 2H_2_ + O_2_) turns water into hydrogen and oxygen [11,36]. Two half reactions—the hydrogen evolution reaction (HER) and the oxygen evolution reaction (OER)—can be used to split water, either through electrolysis or photocatalysis. HER and OER both require an external energy source to be driven because they are energetically uphill reactions. An external electrical potential is used in electrolysis to supply the energy required for the processes to take place [11,36]. A photosensitizer transmits light energy to the catalyst during photocatalysis by absorbing it. A significant obstacle to water splitting is creating HER and OER catalysts that are both effective and affordable. It is known as two-step photocatalytic water splitting because the Z-scheme water splitting system uses two separate photocatalysts to enable the development of H_2_ and O_2_, respectively. The Z-scheme system’s operating concept is shown (Figure 11) [36]. The two main benefits of this system are that it lowers the change in Gibbs free energy, allowing a wider spectrum of visible light to be employed, and that it provides the opportunity to develop semiconductors that can be active for H_2_ or O_2_ evolution, potentially enhancing water degradation performance. Continuous study in this field is essential for accomplishing this aim since efficient water splitting is a significant first step in the development of sustainable and clean energy technologies [36]. The use of metal-based catalysts, metal-free catalysts, and hybrid catalysts are some of the strategies being investigated by researchers to improve the catalytic activity of materials [11,36]. MOFs have also been researched as potential catalysts for HER and OER due to their high surface area, variable pore size, and customized functional groups, [11,36,57].

**Figure 12:**
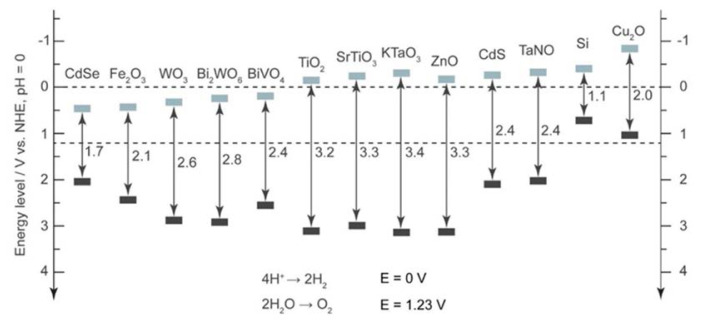
Common semiconductors and their band gap energy [36].

In the process of photocatalytic water splitting, light energy or other types of light are used as the energy source to split water molecules into hydrogen and oxygen [11,36]. Hydrogen may be produced from water, a renewable and abundant resource, using photocatalytic water splitting, which also has the potential to be a sustainable and ecologically benign technology [11,36,57]. Hydrogen can be utilized in fuel cells to generate power or as a clean fuel for transportation. The photocatalyst material absorbs light during the process, creating an excited electron-hole pair that can take part in redox reactions to separate water into its constituent parts. Electron-hole pairs are then employed to reduce protons to create hydrogen fuel gas in the process of driving the oxidation of water molecules to make oxygen gas at the photocatalyst surface. A photocatalyst is employed in photocatalytic water splitting to absorb photons from light and produce electrons and holes, which can subsequently combine with water to make hydrogen and oxygen. A semiconductor substance like TiO_2_, which has a large bandgap and can absorb UV light to produce electrons and holes, can act as the photocatalyst. TiO_2_, ZnO, and other metal oxides, as well as a number of semiconductors and organic compounds, have all been investigated as photocatalytic semiconductor materials for water splitting [11,36,57]. According to Figure 12, which shows the bandgap of typical semiconductors, whole-water splitting must theoretically be catalyzed by a photocatalyst with a minimum bandgap energy of 1.23 eV.

**Figure 13:**
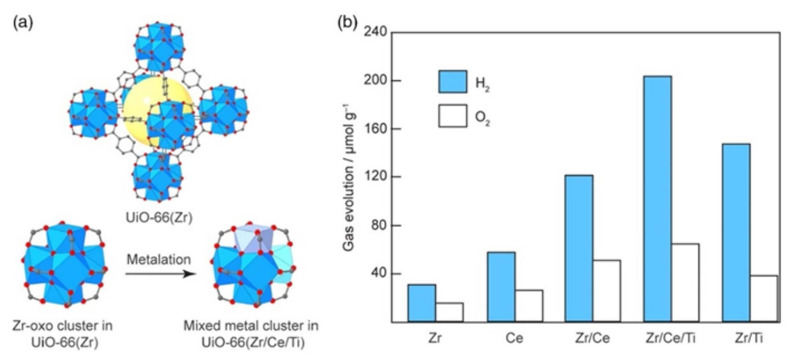
(a) Metalation in UiO-66(Zr) to obtain multimetallic clusters (b) photocatalytic activity of multimetallic clusters in water splitting [36,132].

Excited electrons interact with water molecules at the photocatalyst’s surface during the water splitting photocatalysis process to produce hydrogen ions (H^+^) and hydroxide ions (OH^−^) [11,36]. The excited electrons can then interact with other water molecules to form oxygen gas (O_2_) and additional hydrogen ions in the holes they leave behind. H_2_ can then be created by combining the hydrogen ions with photocatalyst electrons. Research is needed to create more effective materials and optimize the process conditions, as current photocatalysts are not very effective at converting sunlight into hydrogen [11,36,57]. Overall, the production of hydrogen fuel using photocatalytic water splitting has the potential to be environmentally responsible and sustainable [11,36,57]. The effectiveness and stability of the photocatalysts must be improved, and the process’s cost must be decreased, among other obstacles [11,57]. Recently, MOFs have emerged as a promising family of materials for photocatalytic water splitting [11,36,57]. The organic ligands in the MOF structure can aid in improving light absorption and facilitating charge transfer, while the metal nodes of MOFs can act as catalysts for the water-splitting reaction. The structure and content of MOFs can be adjusted to maximize their photocatalytic activity due to their high surface area, which offers a significant number of active sites for catalytic reactions [11,57]. Photosensitizers, which absorb light and transport electrons to the MOF, can functionalize MOFs [11,36,57]. In redox processes, which separate water into hydrogen and oxygen, the excited electrons can then take part [36]. Additionally, metal nanoparticles or metal oxide clusters can be used to functionalize MOFs, acting as catalytic sites for the water splitting reaction [36,57]. The potential of MOFs for photocatalytic water splitting has been demonstrated in a number of studies [36,57]. It has been demonstrated that the zirconium-based MOF, UiO-66, is efficient for photocatalytic water splitting. The substance is stable in both basic and acidic environments, and owing to its high surface area and porosity, it works well as a catalyst for the reaction. UiO-66 and metalated UiO-66 derivatives (Zr/Ti, Zr/Ce, Zr/Ce/Ti, and Ce) were introduced by Melillo and colleagues for photocatalyzing total water splitting under visible-light irradiation [132]. After being tested for 24 h, UiO-66(Zr/Ce/Ti)’s photocatalytic water splitting performance produced results for H_2_ and O_2_ of 210 μmol g^−1^ and 70 μmol g^−1^, respectively. According to Figure 13, the performance order of multimetallic clusters for photocatalytic water splitting is UiO-66(Zr/Ce/Ti) > UiO-66(Zr/Ti) > UiO-66(Zr/Ce). According to this study’s findings, multimetallic clusters are essential for improving the photocatalyst’s ability to split water [132].

**Figure 14:**
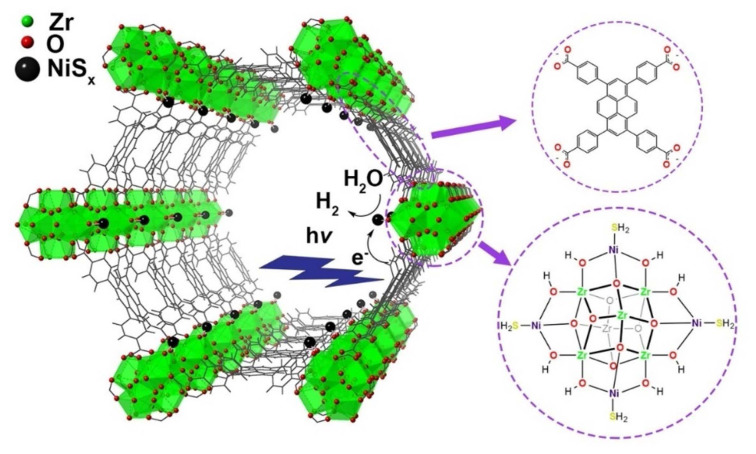
Schematic representation of NU-1000 that has been modified by adding NiS_x_ functionality by atomic layer deposition. In the photocatalytic reaction, a pyrene-based linker acts as a UV sensitizer, transferring an electron to the NiSx-functionalized node, which then reduces protons or water to generate H_2_ [134].

A chromium-based MOF, MIL-101, has been studied for its potential to split water. The substance is a potent catalyst for the reaction due to its high porosity and extensive surface area. The great thermal and chemical stability of MIL-101 also makes it a good option for real-world uses [133]. The zirconium-based MOF, NU-1000, which is seen in Figure 14, has been demonstrated to be efficient for oxidizing water. Since charge carriers must be efficiently captured and transferred in order for the water oxidation reaction to occur, the material has a special structure that makes this possible [134]. Water splitting has been researched for a number of MOFs [135]. The reported MOFs feature large surfaces and distinctive architectures that enable excellent charge carrier capture and transfer, making them efficient catalysts for the process. Water-splitting potential of the copper-based MOF, HKUST-1, has been investigated. The substance has a large surface area and an unusual structure that enable effective charge carrier capture and transfer, making it a powerful catalyst for the reaction [136]. Numerous different MOFs are now being researched for use in water splitting. Table 5 lists MOF photocatalysts for water splitting. As demonstrated, MOFs hold great promise for creating effective and long-lasting photocatalytic devices for water splitting. The creation of MOF-based photocatalysts that may efficiently and sustainably produce hydrogen fuel from water through the water splitting process may be conceivable with extensive research, helping to create a more sustainable energy future.

## 6. Photocatalytic CO_2_ reduction

Using light energy and a photocatalyst material, photocatalytic CO_2_ reduction transforms CO_2_ into valuable chemical compounds [25,43,152–154]. Utilizing a photocatalyst material, which absorbs light to produce an excited electron-hole pair that can engage in redox reactions to change CO_2_ into other chemicals, is required for the procedure [33,43,152]. Metal oxides, semiconductors, and carbon-based materials are a few photocatalytic materials that have been investigated for CO_2_ reduction [25,33,43,153]. Usually, visible or ultraviolet light is used to illuminate these materials, which excites electrons and causes them to travel through the substance. The CO_2_ molecules can be reduced into other compounds when the excited electrons come into contact with the photocatalyst’s surface. Methane, methanol, formic acid, and other valuable compounds are just a few of the products that this method is capable of producing. The reduction of CO_2_ to a product and the oxidation of water to oxygen are the two half-reactions that commonly make up the photocatalytic CO_2_ reduction process. Methane, methanol, or formic acid can be produced by reducing protons (H^+^) with the electrons released during the reduction of CO_2_ [25,33,43]. The type of photocatalyst material, the light wavelength employed, and the reaction circumstances are only a few of the variables that can affect how effective photocatalytic CO_2_ reduction is. There are still a number of issues that need to be resolved, such as low reaction rates, low product selectivity, and poor stability of photocatalysts, despite the potential of photocatalytic CO_2_ reduction as a sustainable way to reduce greenhouse gas emissions and produce valuable chemicals [25,33,152–156]. Addressing these issues and creating more effective and reliable photocatalysts for CO_2_ reduction are the main goals of this field of study.

**Figure 15:**
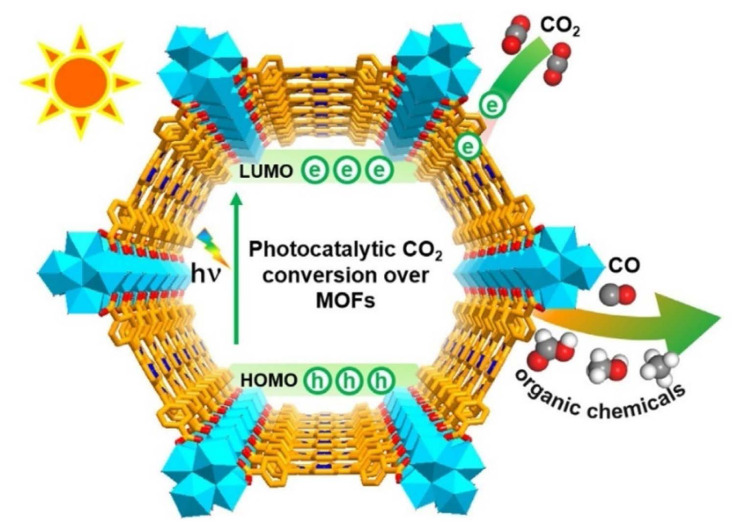
Schematic illustration of photocatalytic CO_2_ reduction over MOFs [154].

**Figure 16:**
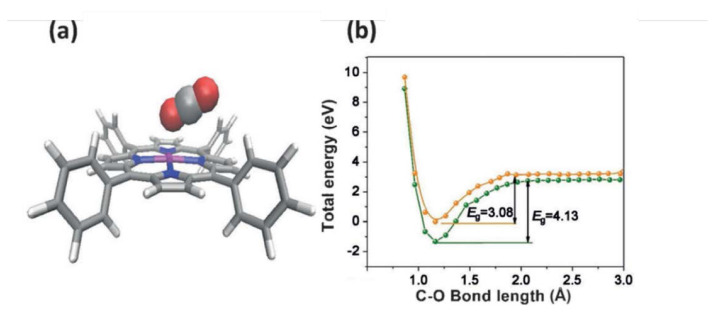
(a) The structure of a porphyrin-Co unit that is capable of CO_2_ adsorption (b) CO_2_ activation energy barrier [157].

**Figure 17:**
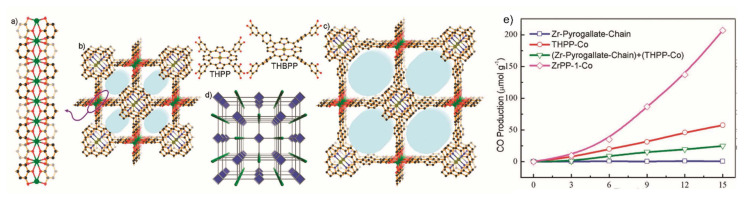
ZrPP-1-Co structure and CO time courses from visible-light-irradiated CO_2_ photoreduction with ZrPP-1-M catalysts [158].

MOFs are potential materials for Carbon Capture and Storage (CCS), which is a key way to reduce CO_2_ emissions. MOFs can be engineered to have a large surface area and particular pore sizes in order to capture and store gases [25,43,27,152–156]. In order to lessen the effects of climate change, it is crucial to reduce CO_2_ emissions, a greenhouse gas that contributes to global warming. CO_2_ can be extracted from the atmosphere or through industrial processes using MOFs, which can then be stored or used to create other beneficial chemicals. One way to lessen carbon dioxide emissions is to use MOFs as catalysts in processes that convert CO_2_ into other molecules. Some MOFs containing metal ions like copper, nickel, or iron can catalyze the transformation of CO_2_ into formic acid or methanol, which can be used as fuels or chemical feedstocks. The conversion of CO_2_ into formic acid or other organic compounds can also be catalyzed by MOFs that contain organic linkers such amino groups [25,33,43,153–156]. Figure 15 [154] included the photocatalytic conversion pathway of CO_2_ into CO and other organic compounds (HCOOH, CH_3_OH, and CH_4_). The other strategy is to use MOFs as adsorbents to remove CO_2_ from the atmosphere or industrial processes. It has been demonstrated that some MOFs have extremely high CO_2_ adsorption capabilities and can pick out CO_2_ from other gases like nitrogen or methane [152–156]. The catalytic methods listed above can be used to store or transform CO_2_ into other valuable compounds after it has been captured. Designing stable, cost-effective MOFs with good selectivity for CO_2_ capture or conversion is the main problem in both situations. To accomplish these objectives, researchers are examining a variety of tactics, such as adding functional groups on the MOF structure or tuning their properties via postsynthetic modification approaches. Researchers have investigated a number of techniques, such as functionalization, pore size modification, and the addition of metal ions with high affinity for CO_2_, to improve the CO_2_ adsorption capacity and selectivity of MOFs [25,33,153]. Additionally, there is ongoing study into how to make MOFs more stable when exposed to flue gas. The work that amine functionalized MIL-101-Cr and also loaded MIL-101 (Cr) with 70% PEI to increase its CO_2_ adsorption capability is one example of employing MOFs for carbon capture. At 25 °C and 1 bar, the resultant amine-modified MOF displayed a high capacity for CO_2_ adsorption; 3.81 mmol/g was achieved on MIL-101 (Cr)-PEI-70. Their adsorption capacities were almost 4.4 times greater than MIL-101 (Cr)’s (0.80 mmol g^−1^) adsorption capacity. A viable contender for postcombustion CO_2_ capture applications, the amine-modified MIL-101(Cr) and MIL-101(Cr)-PEI-70 also showed good stability and recyclability in many adsorption-desorption cycles [152]. MOF-525(Zr)-Co, a porphyrin-based MOF with a singlet Co atom, was described by Ye and colleagues (Figure 16(a), (b)). For the highly selective (36.67 mmol g^−1^h^−1^) light-driven CO_2_-to-CH_4_ reduction, metalloporphyrin coordination frameworks were introduced. As the catalytic centers were atomically scattered, this coordination served as a suitable illustration. In Figure 16(a), porphyrin units released photoexcited electrons to coordinately unsaturated Co centers, and in Figure 16(b), Co centers were active for CO_2_ reduction under the effect of light irradiation [157]. A Zr-MOF with a high CO_2_ uptake capacity and strong photocatalytic activity for the reduction of CO_2_ into CO under visible-light irradiation was created by adding a conjugated porphyrin linker with Co-metallation to ZrPP-1-Co (Figure 17). It was discovered that ZrPP-1-Co’s eclipsed metalloporphyrin array is essential for CO_2_-specific capture and CO_2_-adduct stabilization, which improves photocatalysis. A combination of theoretical calculations and electron spin resonance tests were used in order to investigate the electron traps and reaction kinetics. By isolating the active sites in MOFs, the research by Chen et al. [158] offers a viable method for light-driven CO_2_-to-CO conversion. The CO_2_ adsorption capability of various stable MOF-based photocatalysts is shown in Table 6.

In conclusion, MOFs have promising chances for lowering CO_2_ emissions by capturing carbon and converting it into useful compounds. To make MOFs economically viable for large-scale applications, as well as to improve their efficiency, reduce carbon emissions, and promote environmental sustainability, particularly in the context of CO2 reduction, it is crucial to boost their performance.

## 7. Photocatalytic solar fuel production

Photocatalytic solar fuel production uses a photocatalyst to split water to produce high-energy fuel or fuel precursors [1,2,50]. Semiconducting materials are used in the procedure to absorb solar energy and catalyze the reaction between water and sunlight to produce hydrogen gas [50–52]. This technology has great promise for the generation of sustainable energy since it has the ability to transform solar energy directly into chemical energy that can be stored and used to power houses, cars, and other energy-intensive applications. The photocatalytic process uses a catalyst, often a semiconductor material, to split water molecules into hydrogen and oxygen gas by absorbing photons from sunshine. In addition to being a direct fuel source, H_2_ may also be used to create synthetic fuels like CH_3_OH or hydrocarbons by combining it with CO_2_. Since it does not rely on fossil fuels and does not release harmful pollutants, this method is an appealing way to produce renewable energy. The production of photocatalytic solar fuel comprises a number of processes. First, the photocatalyst absorbs solar light, producing electron-hole pairs. After being transferred to the photocatalyst’s surface, the excited electrons interact with water molecules to create hydrogen gas. The remaining photocatalyst holes are then filled by the oxidation of water, which results in the production of oxygen gas [36,49–51,57]. The synthesis of carbon-based fuels from CO_2_ using a photocatalyst, such as CH_4_ or CH_3_OH, is an illustration of photocatalytic solar fuel production. It is possible to turn carbon dioxide, a greenhouse gas, into a usable fuel through a process known as CO_2_ reduction. There are a number of benefits to using photocatalytic solar fuel production over conventional fuel production techniques. There are no toxic emissions or waste products created during this environmentally favorable and sustainable procedure [1,2,4,50–52]. The procedure is also scalable and applicable to a wide range of fields, including transportation and energy storage [11,36,50,57,153–156]. Despite these difficulties, the advancement of photocatalytic solar fuel production technology holds enormous promise for the future of sustainable and renewable energy. With more study and development, it is feasible that this technology may contribute significantly to supplying the world’s expanding energy needs while lowering carbon emissions and reliance on fossil fuels.

Photocatalytic solar fuel generation in MOFs is a promising approach to converting solar energy into fuels. MOFs are excellent candidates for use in photocatalytic applications, such as solar fuel production, because of their large surface area and customizable characteristics. The use of these porous materials as efficient and affordable catalysts for converting solar energy into fuels such as hydrogen and methane has attracted increasing interest in recent years [1,2,4,50,57]. This field of research involves producing photocatalytic solar fuel in MOFs. MOFs can be utilized as a platform for the immobilization and activation of photosensitizers, which absorb sunlight and transmit the generated energy to catalytic sites inside the MOF [50,51,57]. The energy of the absorbed photons in the visible light spectrum drives this process. These catalytic sites can then speed up the process of converting carbon dioxide or water into fuels that are high in energy, such as H_2_ or hydrocarbons. Incorporating transition metal complexes into the MOF structure is one method for attaining effective photocatalytic solar fuel synthesis. These complexes can act as photosensitizers and catalytic hubs, allowing solar energy to be captured and then used to turn CO_2_ or water into fuels [25,50,153–156]. Another strategy is to employ MOFs as a support and stabilization system for semiconductor materials, such as TiO_2_, which can be used as photocatalysts for the synthesis of solar fuel [36,50–52]. The poor stability and low selectivity of conventional semiconductor photocatalysts can be partially offset by the MOF structure. The potential of MOFs as effective photocatalysts for the synthesis of solar fuel has recently been established through investigations. Furthermore, because MOFs are porous, reactants and products can diffuse through them effectively, enhancing the process’s overall effectiveness.

**Figure 18:**
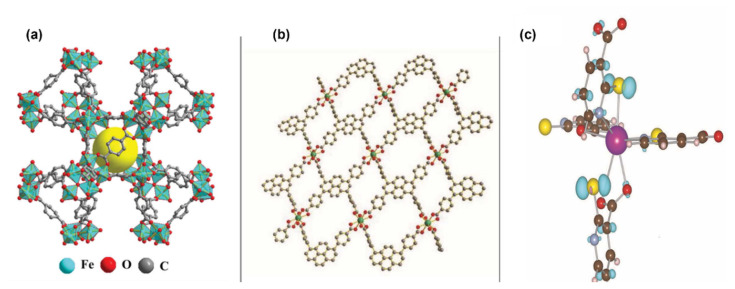
Schmatic crystal structures of photocatalytic MOFs (a) MIL-101(Fe) (b) Cd-TBAPy (c) Bi-mna [169].

MIL-101(Fe) is one type of MOF that is utilized to create oxygen. The very porous MOF MIL-101(Fe) has a large surface area, is adjustable, and is highly porous (Figure 18(a)). It has been demonstrated to have strong photocatalytic activity, with an O_2_ evolution rate reported to reach 219.000 μmolg^−1^ when exposed to visible light [169]. It has also been looked into if other MOFs, including Cd-TBAPy and Bi-mna, could act as photocatalysts for the synthesis of oxygen [141,170] (Figures 18(b) and 18(c)). Table 7 shows the visible light photocatalysis of O_2_ and CH_4_ by certain MOFs.

Overall, photocatalytic solar fuel production in MOFs has the potential to revolutionize the field of renewable energy by offering a sustainable and effective method for fuel production. It also has the ability to generate and store sustainable energy. However, there are still a lot of issues that need to be solved, like enhancing the stability and toughness of MOF catalysts as well as scaling up manufacturing to levels that are feasible.

### 7.1. Photocatalytic H_2_ production

Photocatalytic H_2_ generation has the potential to be employed in renewable energy systems as a clean and sustainable method for manufacturing H_2_ because it employs solar energy, which is a renewable resource, and only produces H_2_ and O_2_ as by-products [175]. A photocatalyst is used in the photocatalytic H_2_ generation method to catalyze a chemical reaction that produces H_2_ from H_2_O. By absorbing light, a photocatalyst in this process creates electron-hole pairs that can drive the redox reactions necessary for water splitting [52]. For this procedure, a variety of photocatalysts such as TiO_2_, ZnO, and other metal oxide semiconductors are utilized. The ability of these substances to absorb light and produce electron-hole pairs, which can subsequently interact with water molecules to form H_2_ and O_2_, is what enables them to do so. The resulting electrons and holes can subsequently be used to decrease H_2_O and oxidize a sacrificed electron donor, producing H_2_ and O_2_, respectively. Increasing the process’s efficiency is one of the difficulties in producing H_2_ by photocatalysis [52,175]. This can be accomplished by enhancing the photocatalyst’s surface area and bandgap energy. By enhancing reaction kinetics and minimizing charge recombination, these accomplishments can contribute to an increase in efficiency. However, a number of issues, including low conversion efficiency and catalyst deterioration over time, continue to plague today’s photocatalytic systems. To solve these problems and create more effective and long-lasting photocatalysts for usage in actual H_2_ production, research is still being done [52,175].

**Figure 19:**
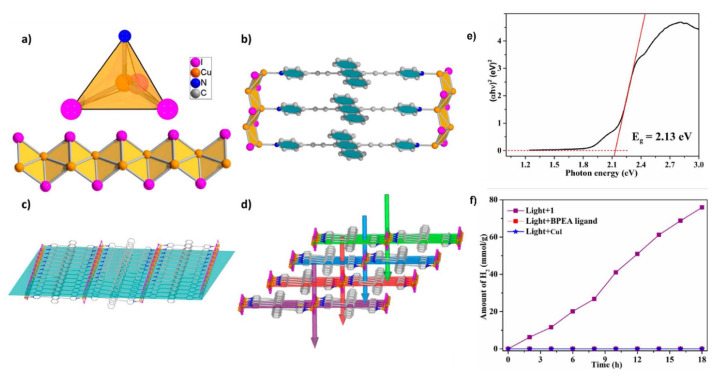
(a) Perspective view of Cu(I) coordination environment (b) The CuI chains are connected via the BPEA ligand, (c) View of 2D sheets (d) the stacking of the 2D layers that the layers are arranged on top of each other to form a 3D structure. (e) The plot of (αhv)^2^ versus photon energy (hv) which may indicate that the material has photovoltaic properties with band gap (Eg = 2.13eV). (f) (a) Comparative photocatalytic H_2_ evolution over 1, BPEA ligand, and CuI [176].

**Figure 20:**
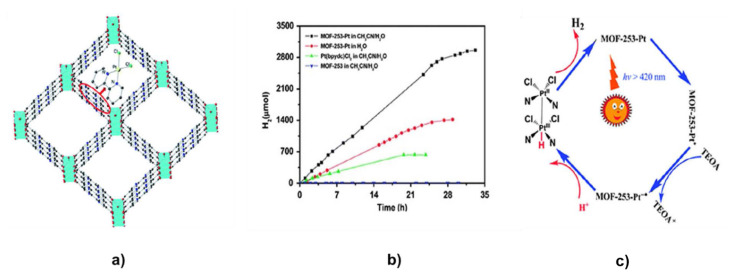
(a) Structure of MOF-253-Pt. (b) Photocatalytic H_2_ production of MOF-253, MOF-253-Pt and Pt(bpydc)Cl_2_. (c) Photocatalytic H_2_ production mechanism of MOF-253-Pt [144].

Due to their distinctive qualities, such as their high surface area, variable pore size, capacity to enable reactions on their surfaces, and chemical stability, MOFs have become a potential class of materials for the synthesis of H_2_ [175]. MOFs can function as electrocatalysts or photocatalysts in the synthesis of H_2_. MOFs can act as electrocatalysts to speed up the electrochemical reduction of protons to H_2_ at the MOF surface. For instance, it has been demonstrated that MOFs containing noble metal nanoparticles (such as Pt or Ru) improve the activity and stability of the catalysts for the generation of H_2_ [52,175]. Functional groups can also be added to MOFs to increase their catalytic activity or selectivity for the synthesis of H_2_. The ability of MOFs to operate as photocatalysts allows them to absorb light, make electron-hole pairs, and then activate the redox process of H_2_O to produce H_2_. By absorbing light and producing charge carriers, MOFs can function as photocatalysts in the photocatalytic synthesis of H_2_ [175]. These charge carriers can subsequently interact with H_2_O molecules to generate H_2_. [Cu_2_I_2_(BPEA)](DMF)_4_ is an illustration of a MOF used to produce H_2_. [Cu_2_I_2_(BPEA)](DMF)_4_ is a porous MOF with a substantial surface area and adjustable properties. With a reported H_2_ evolution rate of up to 4220 μmolg^−1^ under visible light irradiation, it has been demonstrated to have high activity for photocatalytic H_2_ generation (Figure 19) [176]. The potential of other MOFs, such UiO-66-NH_2_ and ZIF-67, as photocatalysts for the synthesis of H_2_ has also been researched [177,178]. A photocatalyst based on MOF has been created by researchers to produce H_2_ from water with solar energy. The MOF, designated MIL-125-NH_2_, was highly effective in splitting water into H_2_ and O_2_ gas using visible light because it was equipped with a Pt nanoparticle co-catalyst. The MIL-125-NH_2_ MOF’s structure permits effective electron transfer and charge separation, and its wide surface area offers a high number of active sites for photocatalytic processes [176–179]. Zhou and colleagues looked into the photocatalytic activity of MOF-253-Pt for H_2_ production. According to the study, H_2_ production rose linearly throughout the first 28 h before gradually reaching its peak at about 30 h. When exposed to visible light, MOF-253-Pt’s photocatalytic process causes the MOF to produce a one-electron-reduced species. MOF-253-Pt’s structure and mechanism are shown in Figure 20(a) and (b) [144]. This method has the ability to produce H_2_ sustainably, which can then be used as a clean, renewable fuel source. Table 8 provides photocatalytic H_2_ synthesis by a few MOFs with H_2_ yield.

Because of their capacity to gather light from a wide range of the electromagnetic spectrum, MOFs have demonstrated potential as photocatalysts. In addition to their catalytic capabilities, MOFs can function as gas separation membranes, which makes it possible to efficiently separate and purify H_2_ from other gases. MOFs are desirable materials for H_2_ separation and purification because they can selectively adsorb H_2_ based on its size and polarity [52,175,176–179]. Due to their high surface area, adjustable characteristics, and capacity to be adjusted for particular purposes, MOFs have generally demonstrated remarkable potential as catalysts for the creation of H_2_ [52,175]. To improve their performance and address problems like stability and cost-effectiveness, more research is necessary.

## 8. Conclusions and outlook

MOFs have shown tremendous potential as photocatalysts for a wide variety of applications in the fields of energy and the environment. These uses include the splitting of water, the reduction of CO_2_, the degradation of pollution, and the creation of H_2._ Because of their adaptable structures, vast surface areas, and functionalization capabilities, MOFs are particularly well-suited for photocatalytic processes. Having said that, there are still a number of difficulties that need to be resolved. For the purpose of their application in practical photocatalytic processes on a broad scale, the development of MOFs with improved stability, high quantum efficiency, and low toxicity will be particularly important. Recently, MOFs as photocatalysts have gained much of interest in the research fields. Thus, a review of the recent advantages of MOF-based materials in photocatalytic applications is highly desirable. Herein, the recent development of MOF-based photocatalysts for energy and environmental applications is presented. This paper aims to provide a comprehensive review of the recent processes in these fields, and also some guidelines for the further development of highly efficient photocatalysts based on MOFs for energy and environmental applications. As a conclusion, the utilization of MOFs as photocatalysts represents a significant step forward in the production of energy solutions that are less harmful to the environment and more sustainable. With continued research and development, MOFs may emerge as more important tools for addressing global problems relating to energy and the environment.

The objectives of this review were centered around exploring the potential of MOFs as photocatalysts in both energetic and environmental applications. Throughout this review, we have delved into the unique properties and characteristics of MOFs that make them promising candidates for harnessing solar energy and addressing environmental challenges. Our primary aim was to assess the photocatalytic efficiency of MOFs and understand their mechanisms in various energy conversion processes. By synthesizing and analyzing the current state-of-the-art research, we have highlighted the substantial progress made in the field and identified key areas for further development. Additionally, we aimed to evaluate the performance of MOFs in promoting sustainable practices and mitigating environmental issues. The review has underscored the significance of MOFs as versatile and efficient photocatalytic materials, showcasing their potential in advancing sustainable energy solutions and environmental remediation.

As we conclude, it becomes evident that MOFs hold tremendous potential for a cleaner and more sustainable future. However, further research is required to address challenges such as stability, scalability, and cost-effectiveness, thereby facilitating their practical implementation in real-world energetic and environmental applications. In summary, this review sheds light on the immense potential of MOFs as photocatalysts, serving as a valuable reference for researchers and scientists working in the fields of energy conversion and environmental sustainability. The ongoing advancements in MOFs as photocatalytic materials will undoubtedly contribute to a greener and more sustainable planet in the coming years.

## Figures and Tables

**Table 1:** Photocatalytic performance of various MOFs for the dyes degradation.

MOFs	Dye Target	Light	Time [min]	Removal [%]	Ref.
MIL-88-A	MB	Vis	50	100	^[68]^
MIL-53(Fe)	MG	Vis	40	100	^[69]^
MIL-53(Fe)	RhB	Vis	40	83	^[69]^
HKUST-1	MG	Vis	85	98	^[70]^
HKUST-1	SO	Vis	85	89	^[70]^
[(CH_3_)_2_NH_2_][ZnL_2_(PW_12_O_40_)]·8DMF·14H_2_O	MB	Vis	17	100	^[71]^
[(CH_3_)_2_NH_2_][ZnL_2_(PW_12_O_40_)]·8DMF·14H_2_O	CV	Vis	24	100	^[71]^
[(CH_3_)_2_NH_2_]3[ZnL_2_(BW_12_O_40_)]·4DMF·4H_2_O	CV	Vis	24	100	^[71]^
[(CH_3_)_2_NH_2_]4[ZnL_2_(CoW_12_O_40_)]·4DMF·7H_2_O	CV	Vis	24	100	^[71]^
[(CH_3_)_2_NH_2_][ZnL_2_(PW_12_O_40_)]·8DMF·14H_2_O	NR	Vis	40	95	^[71]^
[(CH_3_)_2_NH_2_]3[ZnL_2_(BW_12_O_40_)]·4DMF·4H_2_O	NR	Vis	40	95	^[71]^
[(Cd_2_(DDB)(1,3-bimb))·H_2_O]_n_	MO	UV	60	100	^[71]^
[Cd_2_(DDB)(1,3-bmib)(H_2_O)_0.5_)·H_2_O]	MO	UV	60	100	^[71]^
UiO-66(AN)	MO	Vis	90	65	^[72]^
Zr^IV^_6_O_4_(OH)_4_(CO_2_)_12_]	MB	UV/Vis	180	100	^[73]^
[(AgL)(CF_3_SO_3_)]n	MB	UV/Vis	400	100	^[74]^
ST-MOF235	RhB	Vis	40	100	^[75]^
[Cu_2_(L_2_)_2_(CrMo_6_(OH)_5_O_19_)(H_2_O)_2_]·2H_2_O	RhB	UV	350	100	^[76]^
[(Pb(Tab)_2_(bpe))_2_(PF_6_)_4_]	ACBK	UV	60	100	^[77]^
[(Pb(Tab)_2_(bpe))_2_(PF_6_)_4_·1.64AgNO_3_]_n_	ACBK	UV	40	100	^[77]^
[(Pb(Tab)_2_)_2_(PF_6_)_4_]_n_	EBT	UV	24	100	^[77]^
[(Pb(Tab)_2_(bpe))_2_(PF_6_)_4_]_n_	EBT	UV	25	100	^[77]^
[(Pb(Tab)_2_(bpe))_2_(PF_6_)_4_·1.64AgNO_3_]_n_	EBT	UV	15	100	^[77]^
[Mn(phen)_2_(H_2_cpb)]	MB	UV	120	100	^[78]^
[Mn(phen)_2_(H_2_cpb)]	MO	UV	120	100	^[78]^

**Table 2:** Performances of MOFs/MOFs-based composites as photocatalysts for the reduction of Cr (VI) by visible light.

Photocatalyst	Time (min)	Cr (VI) Reduction Efficiency (%)	Ref.
ZnO@ZIF-8	80	100	^[92]^
UiO-66(NH_2_)	80	97	^[90^,^91]^
MIL-101(Fe)	60	100	^[93]^
Pd–Cu/MIL-101	60	100	^[95]^
MIL-53(Fe)	40	100	^[69]^
HPMo@MIL-100(Fe)	8	100	^[93]^
MIL-68	5	100	^[96]^
Au, Pd, Pt@MIL-100	8	100	^[97]^
NH_2_-MIL-125(Ti)/20	60	97	^[98]^
TiO_2_@NH_2_-MIL-88B(Fe)	35	98.6	^[99]^
RGO-UiO-66(NH_2_)	100	100	^[100]^
NNU-36	60	95.3	^[101]^
NH_2_- ZIF-8	180	85	^[102]^
MIL-88B(Fe)	45	100	^[94]^
UiO-66-(OH)_2_	36	80	^[103]^
JLU-MOF60	70	98	^[104]^
AAMNX	70	100	^[105]^
MWCNT/NH_2_-MIL-68(In)	60	100	^[106]^

**Table 3:** Some VOCs and their potential hazards [111].

VOCs	Molecular Structure	Hazard
Benzene	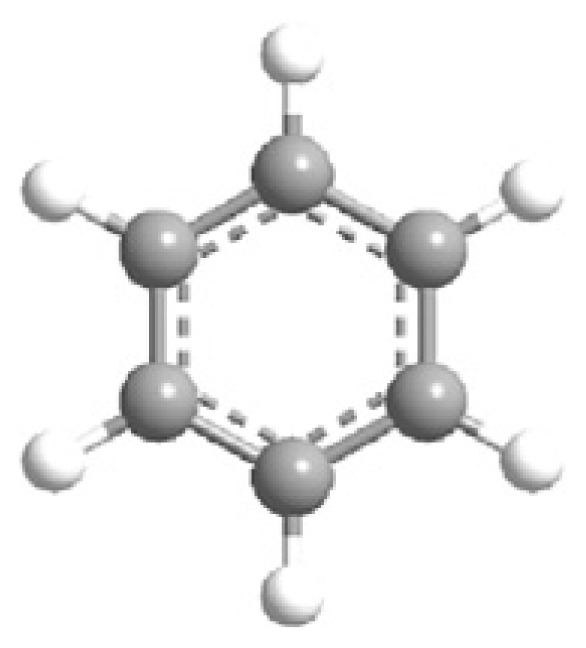	Skin irritation and serious eye irritation
Toluene	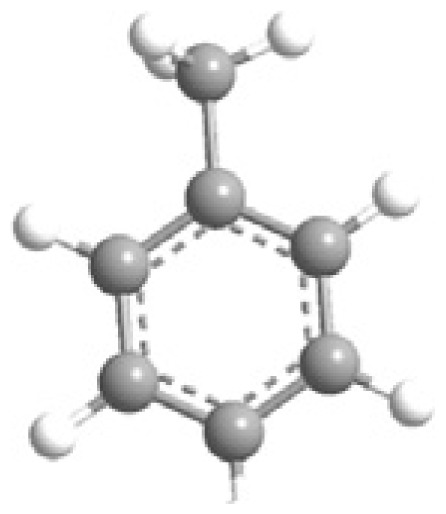	Skin burns, Allergic skin reaction Eye damage
Xylene	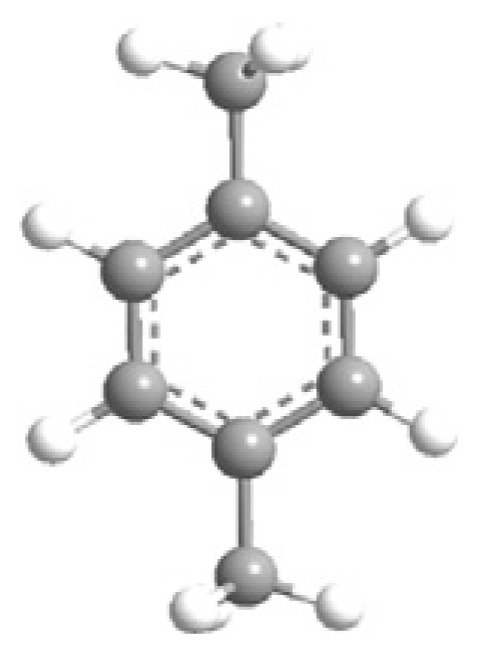	Skin irritation
Chlorobenzene	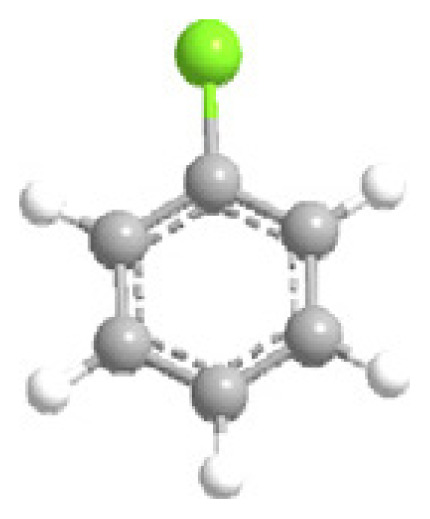	Flammable
Benzyl alcohol	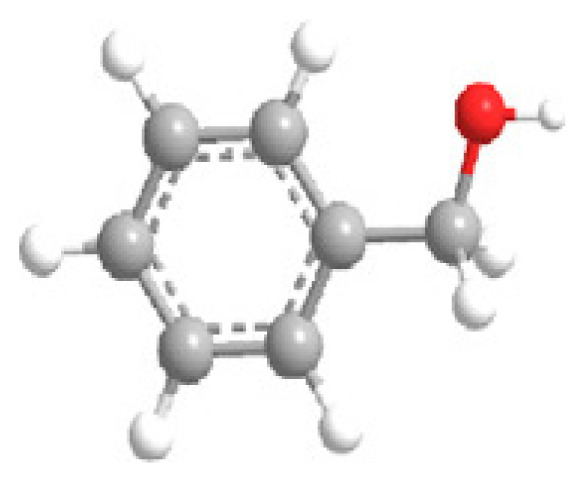	Toxic by inhalation and ingurgitation
Formaldehyde	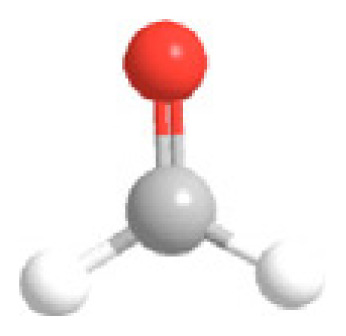	Toxic in contact with skin Toxic if swallowed

The toxicological information of the reaction intermediate products can be found at http://www.hgmsds.com/free-msds ^[111^

**Table 4:** Applications of MOFs and MOFs-based composites for the photocatalysis of VOCs.

VOC Pollutant	MOF-Catalyst	Photodegredation Efficiency [%]	Ref.
Formaldehyde	UiO-66 Ag@Zr-TiO_2_-1000U6	1 h/83.4%	^[117]^
Benzene	MIL-125 N-TiO_2_	8 h/99.1%	^[62]^
Benzene	ZIF-67 Co/NC ZIF-67	6 h/78.9%	^[118]^
Toluene	TiO_2_-MIL-101(Cr)	45 min/94.85%; 90 min/99.7%	^[119]^
Chlorobenzene	P-NH_2_-MIL-125	1 h/100%	^[120]^
Formaldehyde	TiO_2_@NH_2_-MIL-125	48 h/90%	^[121]^
Benzyl alcohol	NH_2_-MIL-125	30 h/94.7%	^[122]^

**Table 5:** MOF photocatalysts for water splitting.

Photocatalyst	Reaction Time (h)	H_2_ (O_2_) production [μmol g^−1^]	Ref.
UiO-66(Zr/Ce/Ti)	24	210 (70)	^[132]^
Al-ATA-Ni	2.5	155 (36)	^[137]^
MIL-101		125	^[133]^
IEF-13	22	46 (23)	^[138]^
MnTD⊂MIL-101(Cr)	1	(10)	^[139]^
Co3.9/MIL-101	1	(175)	^[140]^
Bi-mna	1	(9.64)	^[141]^
(MoS_2_/(-Fe_2_O_3_)/grapheme	3	(2262)	^[142]^
Co_3_O_4_ nanocages	0.5	(85)	^[143]^
MOF-253-Pt	28	28,000	^[144]^
NU-1000-Ni	3	14,400	^[108]^
UiO-66/CdS/RGO	1	13,800	^[145]^
NiMo@MIL-101	2	1480.4	^[133]^
UiO-66(COOH)2/ZnIn_2_S_4_	1	18,794	^[146]^
ZIF-67/Zn0.5Cd0.5S	1	23,265	^[147]^
CdS/MIL-125-NH_2_	3	19,860	^[148]^
Co_3_O_4_/TiO_2_	8	51,200	^[149]^
CdS/Co_9_S_8_	6	61,900	^[150]^
NiS/CdS/h-TiO_2_	4	8000	^[151]^

**Table 6:** CO_2_ adsorption capacity of stable MOFs-based photocatalysts.

Photocatalyst	CO_2_ adsorption (cm^3^/g @273 K)	Ref.
NH_2_-MIL-125(Ti)	132.2	^[159]^
Copper porphyrin MOF	277.4	^[160]^
Au-NH_2_-MIL-125(Ti)	117.4	^[161]^
CPO-27-Mg/TiO_2_	106.59	^[162]^
CPO-27-Mg	250	^[162]^
MIL-101 (Cr)	0.80	^[152]^
MIL-101 (Cr)-PEI-70	3.81	^[152]^
MIL-101(Fe)	26.4	^[163]^
MIL-53(Fe)	13.5	^[163]^
MIL-88(Fe)	10.4	^[163]^
NH_2_-MIL-53(Fe)	20	^[163]^
NH_2_-MIL-101(Fe)	34	^[163]^
NH_2_-MIL-88(Fe)	14.4	^[163]^
NH_2_-UiO-66(Zr)	68	^[164]^
NH_2_-UiO-66(Zr/Ti)	83	^[164]^
TiO_2_ on HKUST-1	49.17	^[165]^
Pt-NH_2_-MIL-125(Ti)	90.2	^[161]^
(NH_2_)/(NH_2_)_2_-UiO-66(Zr)	71	^[164]^
UiO-66	38.4	^[166]^
UiO-66/CNNS	32.7	^[166]^
PCN-222	35	^[167]^
MOF-525	25.3	^[157]^
MOF-525-Zn	33.6	^[157]^
MOF-525-Co	28.1	^[157]^
NNU-28	33.42	^[168]^

**Table 7:** Photocatalytic O_2_ and CH_4_ production of some stable MOFs-based photocatalysts.

Photocatalyst	Fuel yield (μmolg^−1^)	Ref.
ZrPP-1-Co	7.5 (CH_4_)	^[158]^
BIF-101	11.000 (CH_4_)	^[171]^
MOF-525-Co	220.56(CH_4_)	^[157]^
MOF-525-Zn	69.81 (CH_4_)	^[157]^
CsPbBr_3_@ZIF-67	10.537(CH_4_)	^[172]^
MIL-101(Fe)	219.000 (O_2_)	^[169]^
Cd-TBAPy	1634 (O_2_)	^[170]^
Bi-mna	192 (O_2_)	^[141]^
Copper porphyrin MOF	262.6 (CH_3_OH)	^[173]^
Zn_2_GeO_4_/ZIF-8 nanorods	0.22 (CH_3_OH)	^[174]^

**Table 8:** Photocatalytic H_2_ production of some recently published stable MOFs-based photocatalysts.

Photocatalyst	H_2_ yield (μmolg^−1^ h^−1^)	Ref.
20%-MIL-125-(SCH_3_)_2_	3814	^[180]^
Pt@MIL-125/Au	1743	^[181]^
[Cu_2_I_2_(BPEA)](DMF)_4_	4220	^[176]^
PCN-415-NH_2_	594	^[179]^
USTC-8(In)	341.3	^[182]^
ZIF-67	843.7	^[178]^
UiO-66-NH_2_	94	^[177]^
MIL-125-NH_2_	1328	^[179]^
MOF-199/NiMOF-199/Ni	24,400	^[183]^
{[Gd_2_Cu_5_(OH)_2_ (pydc)_6_(H_2_O)_8_]_I8} 500	2050.4	^[184]^
